# Cobalamin Protection against Oxidative Stress in the Acidophilic Iron-oxidizing Bacterium *Leptospirillum* Group II CF-1

**DOI:** 10.3389/fmicb.2016.00748

**Published:** 2016-05-23

**Authors:** Alonso Ferrer, Javier Rivera, Claudia Zapata, Javiera Norambuena, Álvaro Sandoval, Renato Chávez, Omar Orellana, Gloria Levicán

**Affiliations:** ^1^Laboratory of Basic an Applied Microbiology, Department of Biology, Faculty of Chemistry and Biology, University of SantiagoSantiago, Chile; ^2^Program of Cellular and Molecular Biology, Institute of Biomedical Sciences, Faculty of Medicine, University of ChileSantiago, Chile

**Keywords:** *Leptospirillum* group II CF-1, cobalamin, vitamin B_12_, oxidative stress, heavy metals, bioleaching

## Abstract

Members of the genus *Leptospirillum* are aerobic iron-oxidizing bacteria belonging to the phylum *Nitrospira*. They are important members of microbial communities that catalyze the biomining of sulfidic ores, thereby solubilizing metal ions. These microorganisms live under extremely acidic and metal-loaded environments and thus must tolerate high concentrations of reactive oxygen species (ROS). Cobalamin (vitamin B_12_) is a cobalt-containing tetrapyrrole cofactor involved in intramolecular rearrangement reactions and has recently been suggested to be an intracellular antioxidant. In this work, we investigated the effect of the exogenous addition of cobalamin on oxidative stress parameters in *Leptospirillum* group II strain CF-1. Our results revealed that the external supplementation of cobalamin reduces the levels of intracellular ROSs and the damage to biomolecules, and also stimulates the growth and survival of cells exposed to oxidative stress exerted by ferric ion, hydrogen peroxide, chromate and diamide. Furthermore, exposure of strain CF-1 to oxidative stress elicitors resulted in the transcriptional activation of the *cbiA* gene encoding CbiA of the cobalamin biosynthetic pathway. Altogether, these data suggest that cobalamin plays an important role in redox protection of *Leptospirillum* strain CF-1, supporting survival of this microorganism under extremely oxidative environmental conditions. Understanding the mechanisms underlying the protective effect of cobalamin against oxidative stress may help to develop strategies to make biomining processes more effective.

## Introduction

Acidophilic biomining bacteria from the genus *Leptospirillum* are of biotechnological interest due to their use in the recovery of economically important metals from sulfidic ores (Rawlings, 2002; Watling et al., 2014). Members of this genus are chemolithoautotrophic microorganisms that have the ability to oxidize ferrous (Fe^2+^) to ferric (Fe^3+^) iron, a reaction which is highly relevant for the leaching of ores and thus, for the recovery of metals.

*Leptospirillum* strains are able to live in acidic leaching solutions containing elevated concentrations of metals which are toxic to most living organisms. In aerobic environments, iron (Fe^2+^/Fe^3+^) is more soluble and hence, more available under acidic conditions than at neutral pH (Quatrini et al., 2007; Johnson et al., 2012), and thus its bioavailability is usually high. Iron is an essential micronutrient in bacteria and is important for several cellular processes by acting as part of redox centers of various proteins in central metabolism and in electron-transport chains (Carpenter and Payne, 2014). In addition, iron can be used as a primary energy source by many acidophilic microorganisms (Bonnefoy and Holmes, 2012). However, this element can induce damage to cell components due to its ability to generate reactive oxygen species (ROS) through the Fenton or the Haber–Weiss reactions (Papanikolaou and Pantopoulos, 2005; Valko et al., 2005). Besides iron, high concentrations of other heavy metals like copper, nickel and cobalt and of metalloids such as arsenic are usually present in acidic bioleaching environments and may result in the generation of highly oxidative conditions (Ercal et al., 2001; Valko et al., 2005; Slyemi and Bonnefoy, 2012).

Partially reduced ROSs are produced during aerobic respiration (Imlay, 2008). ROS include the superoxide anion ($O_{2}^{•-}$), the hydroxyl radical ($OH^{•}$), and the non-radical oxidant hydrogen peroxide (H_2_O_2_). In addition to its production during aerobic respiration, ROS can be generated in a number of different ways, including exposure to environmental factors such as light, oxidative chemical agents, and metals. In extremely acidic environments, it has been reported that ROS are produced spontaneously on mineral surfaces. Thus, ROS such as H_2_O_2_, $O_{2}^{•-}$, and $OH^{•}$ can be formed on the surface of pyrite with kinetics directly proportional to the surface area of particles (Schoonen et al., 2010; Jones et al., 2013). ROS react with and damage all major molecular components of cells such as DNA, RNA, proteins, lipids and cofactors, thereby having a substantial impact on cell physiology (Cohn et al., 2010; Jones et al., 2013). Additionally, one study using an acidophilic microorganism showed that ROS negatively affect microbial cell growth and the rate of Fe^2+^ oxidation (Jones et al., 2013). These data illustrate that iron-oxidizing and most likely other acidophilic microorganisms are exposed to severe oxidizing conditions that have a negative effect for the cell, and the mechanisms for protection against oxidative stress are thus essential for adaptation and survival in these environments. Although some research has been conducted in this area (Ferrer et al., 2016b), the mechanisms involved in anti-oxidative protection in acidophilic microorganisms are not fully understood.

A meta-proteomic study carried out with the microbial community present in the biofilm of an acid mine drainage (AMD) system, where *Leptospirillum* group II was dominant, revealed that thioredoxins, chaperones and other proteins involved in the defense against oxidative radicals are highly represented, suggesting that they may play an important role in the protection against oxidants in members of the genus *Leptospirillum* (Ram et al., 2005). In addition, a transcriptomic study with material obtained from a natural site that had high concentrations of metals revealed that in *L. ferrooxidans* there is an increased expression of genes that code for proteins involved in DNA repair (RecG and DnaX), carotenoid biosynthesis, and of a putative regulator involved in the response to oxidative stress (Parro et al., 2007). In agreement with these data, the key role of the thioredoxin/thioredoxin reductase (Trx/TR) system in the protection against oxidative-stress was further confirmed for *L. ferriphilum* (Norambuena et al., 2012). Additionally, a bioinformatic analysis revealed that the genome of members of the genus *Leptospirillum* possess genes that encode for several peroxiredoxins, Dyp-peroxidase A, rubrerythrin A, and cytochrome *c* peroxidase (Cárdenas et al., 2012; Contreras et al., 2015). To the best of our knowledge, these bacteria do not carry genes coding for the ROS-scavenging enzymes superoxide dismutase (SOD), catalase (CAT), or for proteins that belong to a glutathione (GSH)-dependent system (Mi et al., 2011; Cárdenas et al., 2012).

Besides the proteins implicated in the response to oxidative-stress and repair of biomolecules, the meta-proteomic study mentioned above revealed the presence of high levels of proteins involved in cobalamin biosynthesis (Ram et al., 2005). It has been suggested that cobalamin could play a role in the adaptation of *Leptospirillum* to this extreme environment and for its establishment in it by increasing the competitiveness during colonization in the later stages of biofilm development (Denef et al., 2010). However, the underlying mechanisms involved in the protective role exerted by cobalamin have not been studied and are not yet understood.

Cobalamin is a cobalt-coordinated tetrapyrrole derived from uroporphyrinogen III, a precursor in the synthesis of heme, siroeheme, and chlorophylls (Raux et al., 2000). In nature, cobalamin is synthesized via a branch of the tetrapyrrole biosynthetic pathway, which involves around 30 enzymes (Martens et al., 2002), and in most bacteria the first intermediate is glutamyl-tRNA (Scott and Roessner, 2002). While many prokaryotes synthesize cobalamin either via oxygen-dependent or oxygen-independent pathways (Scott and Roessner, 2002), other organisms lack the ability to synthesize cobalamin and depend on its uptake from the environment.

In prokaryotes, cobalamin is mainly present in three classes of enzymes: adenosylcobalamin-dependent isomerases, methylcobalamin-dependent methyltransferases, and cobalamin-dependent reductive dehalogenases (Zhang et al., 2009). In addition to its function as an enzyme cofactor, studies using eukaryotic models have revealed that cobalamin also participates in anti-oxidative protection and inflammatory response (Scalabrino et al., 2008; Birch et al., 2009). Furthermore, a protective antioxidative and antiapoptotic role of cobalamin was reported for rat-liver cells exposed to arsenic, where cobalamin restored the activity of the ROS-scavenging enzymes SOD and CAT and increased the level of reduced gluthatione (GSH) (Majumdar et al., 2009, 2012; Chattopadhyay et al., 2012). Furthermore, different forms of cobalamin were shown to have a protective *in vitro* effect against oxidative stress increasing cell viability and reducing cellular damage of endothelial cells exposed to peroxide and superoxide (Birch et al., 2009; Moreira et al., 2011). The facts described above suggest that there is an important link between cobalamin and the response to oxidative-stress in eukaryotic cell lines and animal models. For prokaryotes, an antioxidative role of cobalamin has not been described. Nevertheless, in addition to being important for enzymatic functions, this vitamin directly regulates the transcription of the light-inducible *car* operon involved in carotenogenesis in the non-phototrophic bacterium *Myxococcus xanthus* (Ortiz-Guerrero et al., 2011). In both phototrophic and non-phototrophic organisms, carotenoids serve as protectors against photo-oxidative damage by scavenging harmful radicals, which are formed upon illumination (Ortiz-Guerrero et al., 2011). Thus, from these data an indirect role for cobalamin in the defense against oxidative radicals can be deduced in prokaryotic models. However, a direct effect of this vitamin in regulating ROS concentration and the redox status of the cell has not been demonstrated.

In the present study we investigated the protective effect of cobalamin in *Leptospirillum* strain CF-1 (Lo et al., 2007) in response to oxidative-stress induced by ferric ion, chromate, hydrogen peroxide, and diamide. We evaluated the impact of the exogenous addition of cobalamin on ROS content, oxidative cell damage, and on the activity of antioxidative enzymes. Furthermore, transcriptional expression of genes associated with cobalamin biosynthesis was assessed. Our data revealed the existence of a cobalamin-based mechanism to protect bacterial cells from oxidative damage, and they provide insights into the determinants involved in the tolerance to oxidative-stress in iron-oxidizing acidophilic bioleaching bacteria.

## Materials and Methods

### Culture Condition and Growth Measurement

*Leptospirillum* strain CF-1 (Lo et al., 2007) was obtained from J. Banfield. It was grown in 9K BR medium (Belnap et al., 2010). The cultures were grown aerobically at 37°C with constant stirring at 180 rpm. Bacterial growth was measured by direct microscopic counting by using a modified Neubauer chamber.

### Oxidative Stress Induction

Two liter of *Leptospirillum* CF-1 culture was grown until late exponential phase. Cells were harvested by centrifugation at 9,000 × *g* for 20 min at 15°C. Then cells were resuspended in 25 mL of fresh medium and shaken for 30 min at 37°C. The cells were collected by centrifugation and washed with 10 mM H_2_SO_4_ and resuspended in 25 mL of fresh medium supplemented independently with either 260 mM Fe_2_(SO_4_)_3_ (Sigma–Aldrich), 1 mM H_2_O_2_ (Merck), 6 mM K_2_CrO_4_ (Merck), or 4 mM diamide [1,1′-azobis(*N,N*-dimethylformamide; Sigma–Aldrich)] as oxidative agent for 1 h at 37°C. Samples treated with cobalamin were pre-incubated with 5 nM of cyanocobalamin (Sigma–Aldrich) for 1 h at 37°C in darkness. Cyanocobalamin is by definition vitamin B_12_, and represents the form mainly manufactured by industry (Martens et al., 2002).

### Determination of ROS Levels

The oxidant-sensitive probe H_2_DCFDA (2′,7′-dichlorodihydrofluorescein diacetate) (Davidson et al., 1996) was used to determine the intracellular level of total ROS. For ROS determination, *Leptospirillum* CF-1 cells were washed with 10 mM H_2_SO_4_ and incubated for 30 min in 100 mM potassium phosphate pH 7.4, containing 10 μM final concentration of H_2_DCFDA (from a 1 mM stock solution dissolved in dimethyl sulfoxide). After washing, the cells were suspended in the same buffer, disrupted by sonication, and centrifuged at 18,000 × *g* for 20 min. Aliquots of cell extracts (100 μL) were obtained and the fluorescence intensity was measured using a fluorescence reader (Synergy HT, BioTek) and an excitation at 498 nm. Emission values recorded at 522 nm were normalized to the respective protein concentration. Protein concentration was determined as described by Bradford (1976).

### DNA Extraction

DNA purification was carried out according to standard procedures with some modifications. Cells were collected by centrifugation at 9,000 × *g* for 20 min, washed with 10 mM H_2_SO_4_, resuspended in TE buffer and treated with 2 mg/mL lysozyme (Sigma–Aldrich) for 1 h at 37°C. Chromosomal DNA extraction was carried out according to Sambrook and Russell (2001).

### 8-Hydroxy-2′-deoxyguanosine (8-OHdG) Content

Briefly, 10 μg of DNA were treated with 1 U of DNAse I (Invitrogen) at 37°C for 30 min. After digestion with DNase I, the DNA was denatured for 5 min at 95°C, quickly chilled on ice, and digested with 1 U of nuclease P1 (US Biologicals) for 2 h at 37°C in 20 mM sodium acetate, pH 5.2. After the incubation, the pH was adjusted to 7.5 with 1 M Tris-HCl pH 8.0, and the preparation was treated with 5 U of alkaline phosphatase (FastAP, Thermo Scientific) for 15 min at 37°C. The reaction mixture was centrifuged for 5 min at 6,000 × *g* and the supernatant was used for the measurement of 8-OHdG with the Oxiselect oxidative DNA damage ELISA-kit (Cell Biolabs) following the manufacturer’s instructions.

### Cyclobutane Pyrimidine Dimer (CPD) Content

Cells of strain CF-1 were grown in 125 mL of medium until late exponential phase, collected by centrifugation at 9,000 × *g* for 20 min at 15°C, and incubated during 30 min in fresh medium. Cells were irradiated for 3 min using a UV lamp (256 nm, 8 Watts) at a distance of 30 cm at room temperature. After irradiation, cells were incubated again for 60 min in fresh medium. Then, genomic DNA was purified and CPD content was measured using the OxiSelectTM UV-Induced DNA Damage ELISA Kit (Cell Biolabs, INC) according to the manufacturer’s instructions. Absorbance was measured at 450 nm using a microplate reader (Synergy HT, BioTek).

### Determination of Thiobarbituric Acid-Reactive Substances (TBARS)

Thiobarbituric acid-reactive substances in cell extracts of *Leptospirillum* CF-1 were determined using the Oxyselect TBARS kit (Cell Biolabs Inc.) which detects malondialdehyde (MDA), a byproduct of lipid peroxide oxidation. 2-thiobarbituric acid (TBA) forms adducts with MDA which were measured with a fluorimeter at an excitation of 540 nm and an emission of 590 nm. The concentration of MDA equivalents was determined by using an MDA standard curve.

### Antioxidative Activities

Antioxidative activities were measured in whole-cell extracts prepared according to Norambuena et al. (2012) with some modifications. Bacterial extracts were prepared by ultrasonic disruption in buffer containing 30 mM Tris-HCl pH 8.0, 30 mM NaCl, 1 mM dithiothreitol, followed by centrifugation for 15 min at 30,000 × *g* at 4°C. As a negative control, the activities of a protein extract inactivated at 65°C for 15 min were followed.

#### Thioredoxin (Trx) Activity

Thioredoxin activity was assayed by reduction of disulfides of free chain insulin B by dithiothreitol and measured spectrophotometrically at 650 nm as turbidity formation from the protein precipitation according to Arnér and Holmgren (2001). The assay was carried out with minor modifications at room temperature as described by Norambuena et al. (2012).

#### Thioredoxin Reductase (TR)

Thioredoxin reductase activity was followed by monitoring the reduction of 5,5′-dithiobis-(2-nitrobenzoic acid) (DTNB) at 412 nm according to Lim and Lim (1995) with minor modification as previously described (Norambuena et al., 2012). The reaction was monitored every 30 s for 3 min. The negative control consisted of a protein extract inactivated by heating at 65°C for 15 min.

#### Cytochrome c Peroxidase (CcP)

This activity was assayed as described (Yonetani and Ray, 1966). Briefly, 50 mg of horse heart cytochrome *c* (Merck) were dissolved in 2 mL of 10 mM potassium phosphate pH 7.0 and 1 mM EDTA. To reduce ferricytochrome c, the reaction mixture was incubated with 10 mM sodium dithionite for 2 min. The salt excess was removed by gel filtration in Micro Bio-Spin columns (BioRad) packed with Bio-GelP6 (molecular exclusion limit of 1-6 kDa) (BioRad). Reduced cytochrome *c* was estimated spectrophotometrically at 550 nm. An aliquot of 10 μL was mixed with 490 μL of phosphate buffer pH 7.0 and absorbance was measured at 550 nm. The absorbance of a ferricyanide-oxidized cytochrome *c* was also determined. The percentage of cytochrome *c* reduction was estimated according to Matthis and Erman (1995) using an extinction coefficient (𝜀) of 27.7 mM^-1^ cm^-1^. To measure CcP activity, the reaction mixture (500 μL) contained 10 mM potassium phosphate pH 7.0, 25 mM ferrocytochrome *c*, and 50 μg protein extract. The reaction was started by adding 200 mM H_2_O_2_. The enzyme assay was performed by measuring the oxidation rate of ferrocytochrome *c* every 10 s for 3 min.

#### Superoxide Dismutase

This activity was measured as described by Oberley and Spitz (1984). Xanthine–xanthine oxidase was utilized to generate a superoxide flux. Reduction of nitro blue tetrazolium (NBT) by $O_{2}^{•-}$ to blue formazan was followed at 560 nm, every 30 s for 3.5 min, at room temperature. The rate of NBT reduction in the absence of the extract was used as the reference rate. When increasing amounts of protein (with SOD activity) were added, the rate of NBT reduction was progressively inhibited. The degree of inhibition was expressed as a percentage of the reference rate of NBT reduction when SOD activity was not present. The data were plotted as percentage inhibition versus protein concentration. One unit of activity was defined as that amount of protein necessary to decrease the reference rate to 50% of maximum inhibition. To chelate redox cycling metal ions able to interfere with the reaction, the assay mixture also contained diethylenetriaminepentaacetic acid (DETAPAC). Each l-mL assay tube contained the final concentration of the following reagents: 50 mM potassium phosphate buffer pH 7.8, 1 mM DETAPAC, 60 μM NBT, 0.1 mM xanthine, enough xanthine oxidase to achieve the required reference rate, and 50 μg of protein extract of *Leptospirillum* CF-1. All data were expressed in units of SOD activity per milligram of protein.

### Protein-Free Extract Preparation

Protein-free extract was obtained by filtering the total cell extract through Centricon tubes with a 3 kDa cutoff (Millipore) by centrifugation at 8,500 × *g* for 15 min at 4°C.

### Relative Levels of RNA

#### RNA Isolation and cDNA Synthesis

RNA was isolated using the TRIsure^TM^ reagent (Bioline). DNA was removed by DNase I treatment (Thermo Scientific) according to the manufacturer’s instructions. cDNA synthesis was carried out in 20-μL reaction mixtures containing 1 μg of RNA, 10 pmole of specific primers (**Table 1**), and M-MuLV reverse transcriptase (Thermo Scientific) following the instructions of the provider. cDNA was stored at -80°C until use.

**Table 1:** Primers used for RT-qPCR and qPCR reactions.

Gene	Primer sequence (5′-3′)	Length (bp)	Product
*cobA*	(F)GGGGGAGATCCCTTTGTCTTTG	196	Uroporphyrin III C-methyltransferase/synthase (CobA)


	(R)AGAGTTTTTCCGGATCGTCGTG		
*cbiG*	(F)GAACGCGACTCAGTTCAAGGAATC	192	Cobalt-precorrin 5A hydrolase(CbiG)
	(R)TCCATGTTTTTATCCGGCCG		
*cobU*	(F)CGGATCAGGAAATAAAGGCGAG	197	Adenosylcobinamide kinase/adenosylcobinamide-phosphate guanylyltransferase (CobU)
	(R)GCCTTCCTGCCCCAAAAAG		
*cbiA*	(F)TTCATGAGTGGATTTCCGGGAG	200	Cobyrinic acid a,c-diamide synthase (CbiA)
	(R)GAGGACGGGCAGATCCAGAA		
*rrsB*	(F)TACAAGCTTCCGCTCCTG	113	16S rRNA
	(R)CCGGGCAAAAGTGGTTTACA		

*F: forward. R: reverse*.

#### qPCR Reaction

Primers for all reverse transcription and normal qPCR reactions (**Table 1**) were designed using the available gene sequences of *Leptospirillum* CF-1 (Ferrer et al., 2016a). Then, KAPA SYBR FAST qPCR kits (Kapabiosystems) was used for qPCR amplification according to manufacturer’s instructions. The qPCR conditions were an initial denaturation at 95°C for 5 min, followed by 40 cycles of denaturation (95°C for 30 s), annealing (58°C for 20 s) and extension (72°C for 10 s). All these reactions were performed in a StepOne Real-Time PCR system (Applied Biosystems). The relative abundance of each gene versus a constitutively expressed gene (16S rDNA) was determined. The results were expressed as means of two independent experiments.

### Statistical Analysis

Statistical analysis was performed using the one-way ANOVA test followed by Turkey’s test in GraphPad Prism 5. The differences were considered to be significant at *P* < 0.05.

## Results

### Cobalamin Attenuates Oxidation-Induced ROS Generation

**FIGURE 1:**
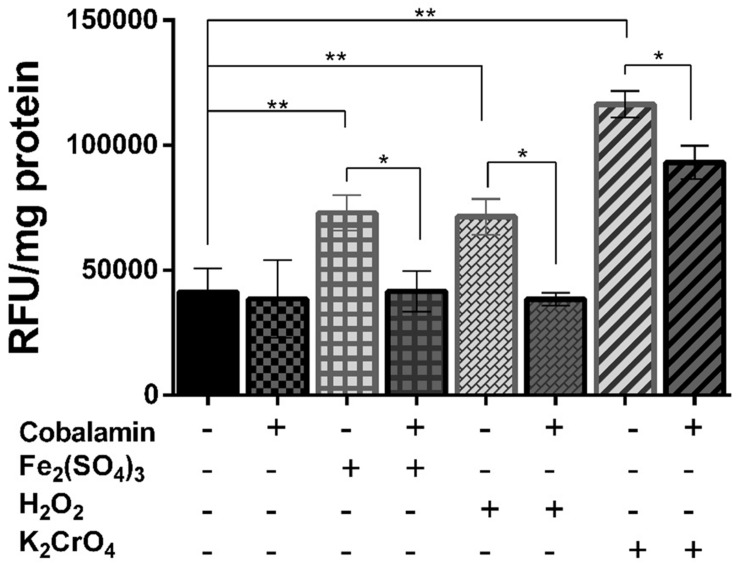
**Effect of cobalamin on ROS generation**. Cytoplasmic ROS content was estimated by measuring activation of the fluorescent-probe H_2_DCFDA in *Leptospirillum* CF-1 cells treated with oxidative agents as indicated under Section “Materials and Methods.” Fluorescence values were expressed as relative fluorescence units (RFU). Bars represent the average of three independent experiments ± standard deviations. ^∗^*P* < 0.05, ^∗∗^*P* < 0.01.

Ferric iron, hydrogen peroxide, and chromate have previously been shown to increase the content of intracellular ROS and to reduce the growth and cell viability of *Leptospirillum ferriphilum* (Cortés et al., 2011). We tested whether cobalamin has a suppressive effect on total ROS generation in *Leptospirillum* strain CF-1 treated with oxidative agents. As shown in **Figure 1**, cells exposed to 260 mM Fe_2_(SO_4_)_3_, 1 mM H_2_O_2_, or 6 mM K_2_CrO_4_ for 60 min showed significantly increased total ROS content (to 176, 172, 282%, respectively) as compared to control cells (100%). Interestingly, a significant reduction of total ROS generation was observed in cells pre-treated for 1 h with 5 nM cyanocobalamin. In cells exposed to ferric iron and hydrogen peroxide, ROS content decreased to levels similar to those detected under control conditions. It should be noted that the concentration of cobalamin required to exert a significant physiological effect was three orders of magnitude lower than those reported to be needed to achieve a similar effect in a eukaryotic cell line model (Birch et al., 2009). These results supported a role of cobalamin as an antioxidant in *Leptospirillum* CF-1 grown under oxidative stress.

Consistent with these results, the decrease in the levels of intracellular ROS correlated with an increase in the growth of cells pre-incubated with 5 nM cyanocobalamin. For example, after 100 h of incubation, exposure to 260 mM Fe_2_(SO_4_)_3_ reduced growth to 28% of control cells, while in cells pre-treated with cobalamin and exposed to the same ROS-elicitor the recovery of cell densities were close (74%) to those of untreated cells. In addition, exposure of strain CF-1 to ferric iron for 60 min lead to a significant reduction in cell viability (45%) compared to non-stressed control cells (100%), while pre-treatment of cells with cobalamin before induction of oxidative stress limited the decrease in cell viability to 70% of the value for the control cells (data not shown).

### Cobalamin Protects Lipids, but Not DNA against Oxidative Damage

**FIGURE 2:**
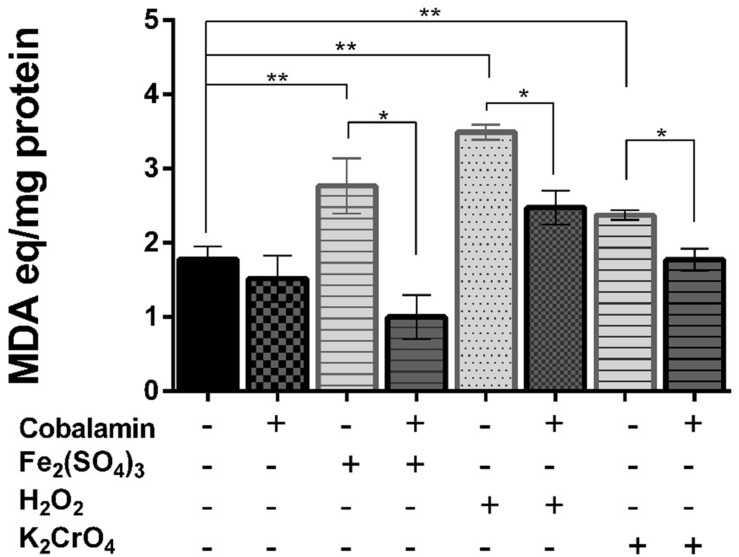
**Effect of cobalamin on lipid peroxidation**. Lipid peroxidation products, expressed as malondialdehyde (MDA) equivalents, were determined as thiobarbituric acid-reactive substances present in crude extracts in *Leptospirillum* CF-1 cells as indicated under Section “Materials and Methods.” Bars represent the average of three independent experiments ± standard deviations. ^∗^*P* < 0.05, ^∗∗^*P* < 0.01.

To determine whether cobalamin plays a role in the protection of biomolecules, we evaluated oxidative damage to lipids and DNA in strain CF-1 exposed to oxidative-stress elicitors. As shown in **Figure 2**, exposure of cells to all of the different oxidative-stress elicitors led to a significant increase in the levels of MDA, as compared to control cells (100%). However, when cells were pre-treated with 5 nM cyanocobalamin for 60 min, the amount of MDA decreased significantly to levels similar or slightly lower (chromate and iron, respectively) than those observed under control conditions. These results are consistent with the ability of cobalamin to reduce the content of intracellular ROS (see above). However, the levels of 8-hydroxydeoxyguanosine, a signature of oxidized DNA, were not significantly different between cells treated with ferric iron in the presence or absence of cobalamin (∼125% of untreated cells, data not shown). Thus, it seems that the protective effect of cobalamin is not a general scavenging system for any ROS that is generated. Similarly, we did not detect a significant effect on the content of cyclobutane pyrimidine dimer (CPD) upon exposure to UV light, showing that cobalamin did not exert direct protection against oxidation of DNA.

### Cobalamin Increases the Activity of Protective Antioxidant Proteins

As previously described, *Leptospirillum* group II possesses a thioredoxin-based thiol/disulfide system (Ram et al., 2005; Norambuena et al., 2012). In addition, inspecting the genome sequence of *Leptospirillum* CF-1 allowed us to identify genes encoding for several peroxidases (cytochrome *c* peroxidase, rubrerythrin A, and Dyp peroxidase). In order to evaluate whether cobalamin exerts a role in the activation of anti-oxidative enzymes in *Leptospirillum* CF-1, we measured the activity of cytochrome *c* peroxidase (CcP) and of the thioredoxin/thioredoxin reductase (Trx/TR) system. Since superoxide scavenging enzymes are considered ubiquitous in aerobic organisms, we also tested cells for this enzymatic activity, although no canonical SOD gene was identified in the genome of strain CF-1.

#### Thioredoxin System

**FIGURE 3:**
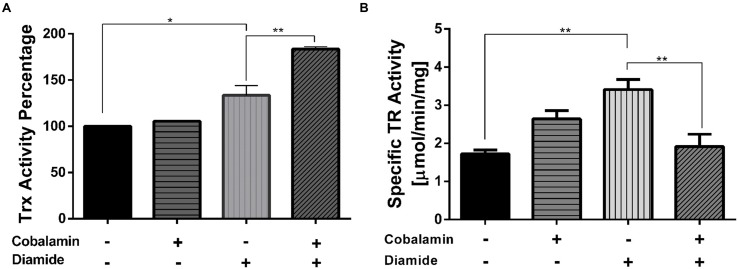
**Effect of cobalamin on thioredoxin and thioredoxin reductase activity**. To measure Trx activity **(A)**, the reduction of the alpha-chain of insulin was monitored at 650 nm as described under Section “Materials and Methods.” TR activity **(B)** was monitored by following the reduction of 5,5′-dithiobis-(2-nitrobenzoic acid) (DTNB) at 412 nm. The activity in the control reaction corresponds to 100%. Data represent the average of two independent experiments (lines on top of bars indicate value ranges). ^∗^*P* < 0.05, ^∗∗^*P* < 0.01.

As is shown in **Figure 3A**, exposure of strain CF-1 to the disulfide-stress elicitor diamide led to an increase in thioredoxin activity, as compared to control cells. Interestingly, pre-treatment with 5 nM cyanocobalamin for 60 min led to a significant increase in thioredoxin activity in cells that were exposed to diamide (184%), but not in cells grown under the control condition. A similar trend was also observed when cells were exposed to ferric iron as an elicitor of oxidative-stress (data not shown).

To evaluate whether the increase in Trx activity involved a coordinated increase in thioredoxin reductase activity (TR), TR activity was measured using the DTNB-reduction method in whole cellular extracts. Exogenous addition of cobalamin resulted in decreased TR activity in cells exposed to diamide for 30 min (**Figure 3B**). Thus, changes in TR activity could not directly explain the increase in Trx activity described above. Consequently, the marked effect on Trx activity upon treatment of cells with diamide seems to be the result of a different mechanism.

#### Cytochrome c Peroxidase

**FIGURE 4:**
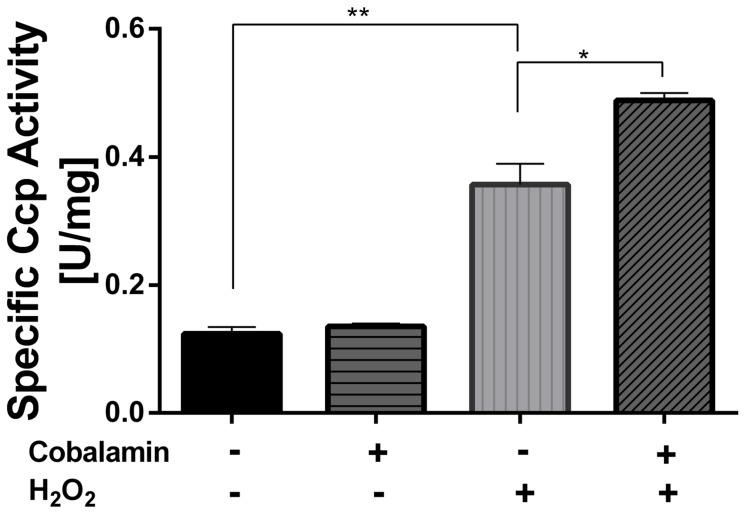
**Effect of cobalamin on CcP activity**. The activity was followed spectrophotometrically at 550 nm as indicated under Section “Materials and Methods.” Data represent the average of two independent experiments (lines on top of bars indicate value ranges). One unit (U) is defined as the amount of enzyme required to oxidize 1 μmol of ferrocytochrome *c* per min. ^∗^*P* < 0.05, ^∗∗^*P* < 0.01.

Cytochrome c peroxidase activity was measured in whole-cell extracts following the oxidation of ferrocytochrome *c* (**Figure 4**). In agreement with the bioinformatic analysis mentioned above, CcP activity was in fact detected in whole-cell extracts of this strain. In addition, CcP activity was significantly increased when cells were exposed to 1 mM hydrogen peroxide for 30 min (288%), as compared to control cells (100%). Pre-treatment of cells with 5 nM cyanocobalamin further increased CcP activity in cells exposed to hydrogen peroxide (394%). It is important to note that the activating effect of cobalamin was only observed in cells that had been exposed to elicitors of oxidative stress and not in cells treated solely with cobalamin. A similar trend was observed in Fe_2_(SO_4_)_3_-stressed cells (data not shown).

#### Superoxide Dismutase-Like Activity

**FIGURE 5:**
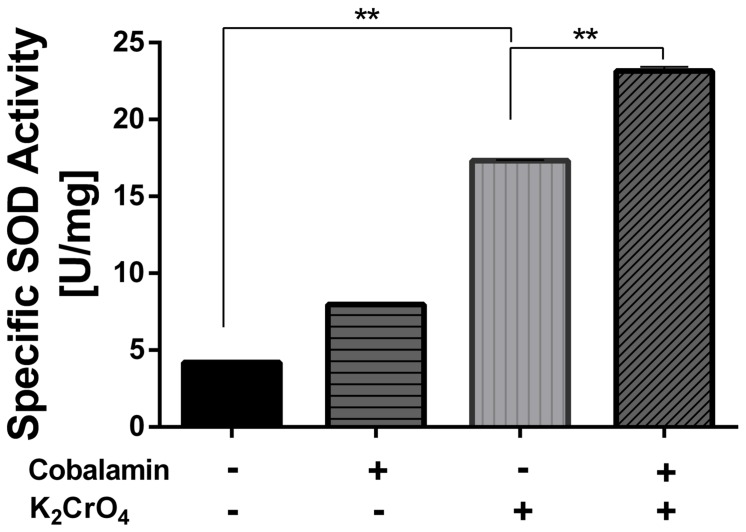
**Effect of cobalamin on superoxide dismutase activity**. The activity was measured by following the inhibition of NBT-reduction at 560 nm as described under Section “Materials and Methods.” Data represent the average of two independent experiments (lines on top of bars indicate value ranges). One unit (U) is defined as the amount of enzyme necessary to decrease the reference rate to 50% of maximum inhibition. ^∗∗^*P* < 0.01.

The activity was evaluated using a xanthine oxidase-based superoxide generating system. After 30 min of exposure to 6 mM K_2_CrO_4_, SOD activity did not exhibit a significant change. In contrast, SOD activity increased dramatically after a 60 min exposure to the oxidative-stress elicitor (413%), as compared to the control (100%) (**Figure 5**). Like the other antioxidative activities that were analyzed, pre-treatment with cobalamin further increased SOD activity up to a 552% (*P* < 0.05). However, unlike Trx and CcP activities, SOD activity increased considerably in cells pre-incubated with cobalamin in both the presence or absence of oxidative stress elicitors. In addition, consistent with what was observed for CcP activity (see above), a similar activating effect of cyanocobalamin on SOD activity was observed in cells stressed with 260 mM Fe_2_(SO_4_)_3_ (data not shown).

It is surprising that although superoxide-anion scavenger activity was detected, no genes encoding for SOD enzymes were found in the genome sequence of strain CF-1. This fact suggests the existence of an alternative repertoire of genes encoding either non-canonical catalysts of $O_{2}^{•-}$ dismutation, or of enzymes involved in the biosynthesis of non-catalytic scavengers (Miriyala et al., 2012). Thus, we were interested in determining if the superoxide-anion radical-scavenger activity was present in the protein-enriched or protein–free fractions of cells exposed to oxidative conditions. In order to obtain these fractions, the extract derived from ferric iron-stressed cells was passed through a 3 kDa cut-off spin filter. The results (**Figure 1**, Supplementary Material) showed that the superoxide-anion scavenger activity is present in the protein-free fraction obtained after filtering the whole extract. Supporting this result, the activity was detected in whole-cell extracts after both protease and heat treatment (data not shown), strongly suggesting that the SOD-mimetic activity of strain CF-1 is determined by a non-proteinaceous metabolite.

### Effect of Oxidative Stress on the Endogenous Cobalamin-Biosynthesis Pathway

**FIGURE 6:**
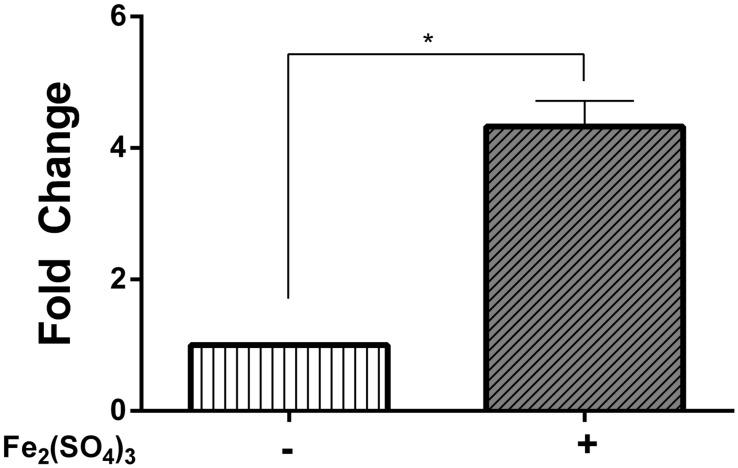
**Relative mRNA levels of the *cbiA* gene in *Leptospirillum* CF-1**. Bacteria were treated with 260 Fe_2_(SO_4_)_3_ for 30 min. Data were normalized by the 16S rRNA. Data represent the average of two independent experiments (bar indicates the value range). ^∗^*P* < 0.05.

Since cobalamin exerted a protective effect on cells exposed to oxidative-stress elicitors, it raised the question as to whether there is an increase in the activity of the cobalamin-biosynthetic pathway when *Leptospirillum* CF-1 is exposed to oxidative conditions. The inspection of the genome of strain CF-1 revealed the existence of 19 genes involved in the *de novo* non-oxygen-requiring biosynthetic route of cobalamin from 5-aminolevulinic acid (Supplementary Figure S2). In order to evaluate the activity of the biosynthetic pathway upon exposure to oxidative compounds, we determined the mRNA level of key genes associated with different parts of the pathway. Total RNA was isolated from strain CF-1 exposed to 260 mM Fe_2_(SO_4_)_3_ and the mRNA level of genes encoding for CobA, CbiG, CbiA, and CobU, as well as of one housekeeping gene (*rrsB*) was quantified by qRT-PCR. It should be noted that no significant changes were observed in the levels of the *rrsB* mRNA from strain CF-1 under any of the experimental conditions that were assayed (data not shown). The real-time PCR data showed that the four genes were, in fact, transcribed in strain CF-1 exposed to ferric iron. The genes *cobA*, *cbiG*, and *cobU* did not show significant changes in their transcript level between treated and untreated cells. However, the *cbiA* gene encoding for cobyrinic acid a,c-diamide synthase was significantly up-regulated in response to ferric iron-induced stress after a 30 min exposure (**Figure 6**). This result is in agreement with previous reports showing that the *cbiA* gene is upregulated in *Escherichia coli*, *Salmonella* Typhimurium, and *Dehalococcoides mccartyi* in response to short-term cobalamin deprivation (Rodionov et al., 2003; Men et al., 2014). Therefore, it can be presumed that in cells cultured under oxidative stress, the cobalamin biosynthetic route is activated as a strategy to contribute to alleviating the stress conditions.

## Discussion

Acidic bioleaching environments are considered as highly oxidative where microorganisms are exposed to elevated concentrations of ROS (Cárdenas et al., 2012; Ferrer et al., 2016b). Thus, oxidative stress represents one of the greatest selective pressures imposed on acidophilic microorganisms. Members of the genus *Leptospirillum* grow in these extreme environments, however, they lack the main canonical enzymatic activities involved in antioxidant responses, namely SOD, CAT, and the glutathione system (Cárdenas et al., 2012), suggesting that alternative molecular mechanisms to maintain redox homeostasis might be present in these extremophilic microorganisms. In this study we showed that externally added cobalamin is able to mitigate oxidative stress in *Leptospirillum* CF-1, as judged by its effect on modulating the concentration of intracellular ROS and lipid damage of cells exposed to various oxidative-stress elicitors. In addition, cells exposed to cobalamin exhibited significantly enhanced cell density and viability under oxidative conditions. In agreement with these facts, the mRNA level of a gene associated with the *de novo* biosynthesis of cobalamin showed a significant increase after exposure to oxidative conditions, suggesting the up-regulation of this metabolic pathway. Therefore, we propose that cobalamin has a role in preventing oxidative-stress that could be relevant under the extreme conditions encountered in acidic environments.

Previously, it has been reported that reduced forms of cobalamin can directly dismutate the superoxide ion at rates approaching those exhibited by SOD enzymes (Suarez-Moreira et al., 2009). This finding is particularly significant in view of the observation that SOD-like activity could be detected in protein-free fractions of strain CF-1. For other organisms, a number of metabolites such as thiamine, pyrroloquinoline-quinone, or Mn/Fe-porphyrin derivatives with potent $O_{2}^{•-}$ scavenger activity have been reported (Jung and Kim, 2003; Misra et al., 2004; Miriyala et al., 2012), and many of them are also potentially efficient scavengers of other reactive species such as peroxynitrite, the peroxyl radical, or H_2_O_2_ (Batinić-Haberle et al., 2010). It is tempting to speculate that in CF-1 cells, cobalamin may efficiently replace the SOD enzyme and play a major role in the detoxification of $O_{2}^{•-}$, and perhaps, of other reactive species. Based on our findings, this effect would be achieved at low concentrations compared to other molecules that have been reported as having a non-catalytic antioxidative activity, such as glutathione (Imlay, 2008). Thus, the potential cobalamin-based mechanism to scavenge $O_{2}^{•-}$ could require the occurrence of molecular component(s) highly efficient in the maintenance of the redox status of the vitamin, allowing that its scavenging activity proceeds at high rates. However, at this point, the existence of other low molecular-weight compounds with SOD-mimetic activity in leptospirilli cannot be ruled out.

Cobalamin is known to be involved in the metabolism of methionine, a well-known antioxidant, by being a cofactor of methionine synthase (Levine et al., 2000; Halsted, 2013). The genome analysis of strain CF-1 revealed the presence of a gene that probably encodes a cobalamin-dependent methionine synthase (Ferrer et al., 2016a), suggesting that in this microorganism cobalamin could have an antioxidant activity through restoring methionine metabolism. Moreover, methionine is a precursor for *S*-adenosylmethionine which also has antioxidative properties as it is able to chelate Fe^2+^, thereby significantly preventing the occurrence of the Fenton reaction (Caro and Cederbaum, 2004). Thus, the mode of action of cobalamin in *Leptospirillum* CF-1 may proceed through different mechanisms that involve both a direct role as a ROS-scavenger system and indirect roles by participating in methionine metabolism.

In this work we observed an effect of cobalamin on restoring and even increasing the activity of antioxidative proteins such as CcP and thioredoxins. In agreement with these results, a similar effect was observed in rats, where pre-incubation with cobalamin restored the activity of SOD and CAT which were significantly inhibited in individuals treated with arsenic. Similarly, supplementing cobalamin to these rats could significantly restore the level of hepatic mitochondrial GSH as compared with an arsenic-treated group (Majumdar et al., 2012). Along the same line, it has been reported that lymphocytes from patients with cobalamin deficiency show a decrease in the pool of total and reduced glutathione (Pastore et al., 2012).

In prokaryotes, besides its primary role as an enzyme cofactor, cobalamin is also involved in the transcriptional regulation of genes related to carotenoid biosynthesis. In this process, gene regulation is based on the binding of cobalamin to the CarH repressor in a light-dependent manner (Ortiz-Guerrero et al., 2011). Interestingly, a role for cobalamin in the regulation of carotenogenesis has also been suggested in *Streptomyces coelicolor* (Takano et al., 2006) and in extremophilic bacteria of the genera *Deinococcus* and *Thermus* (Tian and Hua, 2010). Also, this metabolite may directly regulate gene expression via a riboswitch (Henkin, 2008). Some examples of genes regulated via a cobalamin-riboswitch include the genes for methionine synthase in *Bacillus clausii* and *Mycobacterium tuberculosis*, and genes of the *btu* operon in *Escherichia coli*, which are involved in cobalamin uptake. These data support the idea that regulation of antioxidative enzymes by cobalamin in strain CF-1 may occur at the level of mRNA abundance of the corresponding genes. Thus, further studies addressed to identify cobalamin-induced changes of the transcriptome and proteome of strain CF-1 would undoubtedly help us to better understand how cobalamin stimulates the activity of the antioxidative proteins.

Noticeably, the antioxidative role of cobalamin that was detected correlates with increased transcript levels of the *cbiA* gene after exposure to oxidative-stress elicitors. Therefore, we speculate that the redox status of cells may be involved in regulating the activity of the cobalamin-biosynthetic pathway. Whether this leads to an increased concentration of intracellular cobalamin should be addressed. On the other hand, it will also be interesting to address if special modifications of the axial ligand of cobalamin (Hannibal et al., 2008, 2013; Yan et al., 2015) are relevant and are determinants of the antioxidative properties that have been described for this vitamin.

Finally, it is a well-known fact that cobalamin biosynthesis is confined to Archaea and some Bacteria. Since it is a highly complex and energy-consuming process with about 30 enzymatic steps (Martens et al., 2002; Scott and Roessner, 2002), questions arise about the driving forces that contributed to select a cobalamin-based mechanism to restore the redox balance within the cell and to protect cells against oxidative damage. The evidence presented herein places cobalamin as part of the cellular oxidative-stress defense scheme of *Leptospirillum* CF-1, and likely of other members of this genus. In light of the facts discussed above, it is conceivable to postulate that the multi-target effect of cobalamin may contribute to maintain the redox balance under highly oxidizing conditions, leading to a global activation of cellular components that participate in the response to oxidative-stress. Thus, cobalamin might provide a specific advantage in extremely acidic and highly metal-loaded environments by increasing the tolerance and fitness of these microorganisms. Whether the cobalamin-based system is confined to leptospirilli or is a niche-specific adaptation of acidophilic microorganisms is still an open question.

## Author Contributions

GL and OO: conceived and designed the experiments. AF, JR, CZ, JN, AS: performed the experiments. AF and RC: analyzed the data. GL, AF, and OO: wrote the paper.

## Conflict of Interest Statement

The authors declare that the research was conducted in the absence of any commercial or financial relationships that could be construed as a potential conflict of interest.
