# Supplementary figures and images for: Cobalamin Protection against Oxidative Stress in the Acidophilic Iron-oxidizing Bacterium Leptospirillum Group II CF-1
Source: Front Microbiol. 2016 May 23;7:748. doi: 10.3389/fmicb.2016.00748 (PMC4876134; doi:10.3389/fmicb.2016.00748)

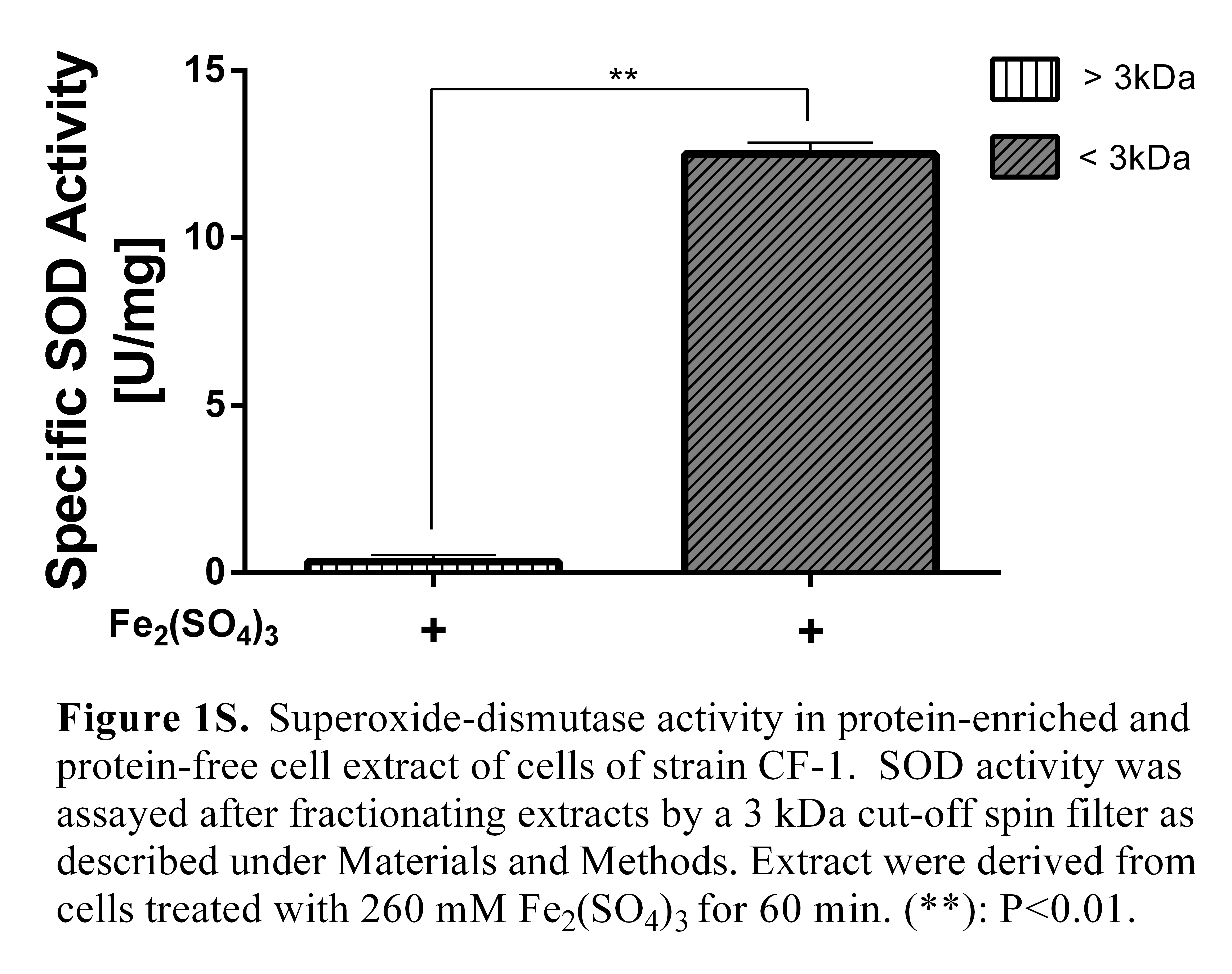

Supplement: Supplementary file 1 [file Image_1.JPEG]
